# 1552. HIV Pre-Exposure Prophylaxis Awareness, Uptake, and Persistence Among People at Risk for HIV in Two Communities in Western Kenya

**DOI:** 10.1093/ofid/ofad500.1387

**Published:** 2023-11-27

**Authors:** Matthew L Romo, Glenna Schluck, Britt Gayle, Josphat Kosgei, Christine Akoth, Rael Bor, Deborah Langat, Margaret Yacovone, Julie A Ake, Fred Sawe, Trevor A Crowell

**Affiliations:** US Military HIV Research Program, Walter Reed Army Institute of Research; Henry M. Jackson Foundation for the Advancement of Military Medicine, Bethesda, Maryland; US Military HIV Research Program, Walter Reed Army Institute of Research; Henry M. Jackson Foundation for the Advancement of Military Medicine, Bethesda, Maryland; US Military HIV Research Program, Walter Reed Army Institute of Research; Henry M. Jackson Foundation for the Advancement of Military Medicine, Bethesda, Maryland; HJF Medical Research International; U.S. Army Medical Research Directorate–Africa, Kericho, Rift Valley, Kenya; HJF Medical Research International; U.S. Army Medical Research Directorate–Africa, Kericho, Rift Valley, Kenya; HJF Medical Research International; U.S. Army Medical Research Directorate–Africa, Kericho, Rift Valley, Kenya; HJF Medical Research International; U.S. Army Medical Research Directorate–Africa, Kericho, Rift Valley, Kenya; National Institute of Allergy and Infectious Diseases, National Institutes of Health, Rockville, Maryland; Walter Reed Army Institute of Research, Silver Spring, Maryland; HJF Medical Research International; U.S. Army Medical Research Directorate–Africa, Kericho, Rift Valley, Kenya; Henry M. Jackson Foundation for the Advancement of Military Medicine, Bethesda, Maryland

## Abstract

**Background:**

Despite the increasing availability of evidenced-based HIV prevention tools like pre-exposure prophylaxis (PrEP), HIV incidence remains disproportionately high in sub-Saharan Africa, especially among key populations. To inform the ongoing scale-up of PrEP in Kenya, we examined PrEP awareness, uptake, and persistence among participants at enrollment into an HIV incidence cohort.

**Methods:**

For these cross-sectional analyses, we used interim data from the screening and enrollment visits for the ongoing longitudinal Multinational Observational Cohort of HIV and Other Infections (MOCHI) in Homa Bay and Kericho, Kenya. Participants were at risk for HIV based on recent history of sexually transmitted infection, transactional sex, condomless sex, and/or injection drug use. At enrollment, participants were asked about PrEP awareness and use, agreement with statements related to concerns about PrEP and interest in different types of PrEP, and reasons for stopping PrEP.

**Results:**

Between December 2021 and March 2023, 384 participants were enrolled (213 in Homa Bay, 171 in Kericho). The mean age was 22 years (SD 4), 302 (79%) were female, and 242 (64%) were sex workers. All participants were eligible for PrEP based on national guidelines, 262 (68%) had heard of PrEP, 65 (17%) had taken PrEP, and 20 (5%) were currently using PrEP, with greater PrEP awareness and uptake in Homa Bay vs. Kericho **(figure 1)**. Of the 197 participants who were aware of PrEP but never used it, 100 (51%) were concerned about side effects of taking a medication every day, 70 (36%) were concerned about PrEP costs, and 65 (33%) did not perceive themselves to be at risk for HIV. Among those aware of PrEP but never used it,115 (58%) had interest in daily oral PrEP and 146 (74%) had interest in long-acting injectable PrEP. Of the 65 participants who had taken PrEP, 36 (55%) reported ever stopping it, with ‘trusting my partner’ as the most common reason **(figure 2)**.

**Figure:**
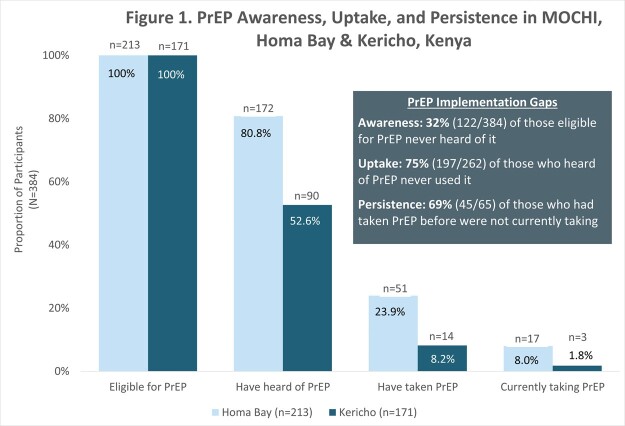

**Figure:**
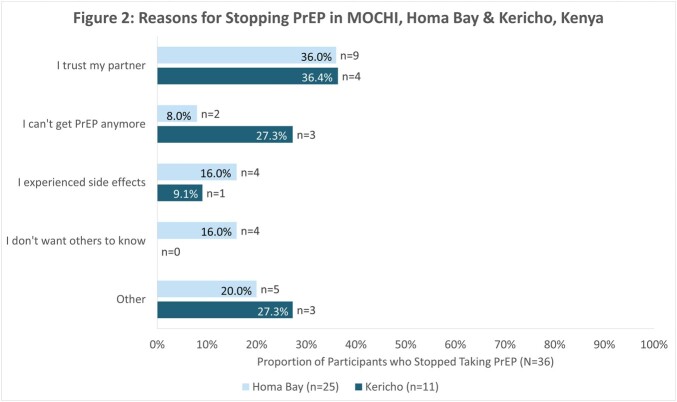

**Conclusion:**

We identified substantial gaps in PrEP awareness, uptake, and persistence among individuals in Kenya who were eligible for PrEP. Potential strategies to improve PrEP engagement include education about HIV risk, addressing concerns about daily oral PrEP, and implementing additional options including long-acting injectable cabotegravir.

**Disclosures:**

**All Authors**: No reported disclosures

